# Why is prone positioning so unpopular?

**DOI:** 10.1186/s40560-016-0194-8

**Published:** 2016-11-25

**Authors:** Jason Chertoff

**Affiliations:** Division of Pulmonary and Critical Care Medicine, Department of Internal Medicine, University of Florida College of Medicine, 1600 SW Archer Road, Gainesville, FL 32608 USA

## Abstract

Recent studies have shown acute respiratory distress syndrome (ARDS) to be underdiagnosed and inadequately treated, as evidenced by underutilization of low-tidal volume ventilation. Despite a proven survival benefit in patients with severe ARDS, studies have also shown underutilization of prone positioning. Many questions persist as to the reasons for prone positioning’s unpopularity. Additional studies are required to uncover the causes of this prone positioning underutilization phenomenon.

## Commentary

Recently, an international, multicenter, prospective cohort, “The Large Observational Study to Understand the Global Impact of Severe Acute Respiratory Failure” (LUNG SAFE) showed acute respiratory distress syndrome (ARDS) to be widely unrecognized and inadequately treated [1]. Shockingly, the study reported that clinical recognition of ARDS ranged from 51.3 in mild to 78.5% in severe ARDS, while less than two-thirds of ARDS patients received tidal volumes of 8 ml/kg or less, and only 16.3% with severe ARDS were prone positioned [1]. After reviewing these results, I initially focused on the surprisingly poor utilization rate of low-tidal volume ventilation, an intervention repeatedly proven to have significant survival benefit and deemed by most to be a cornerstone in ARDS management [2]. Even more alarming than this suboptimal use of low-tidal volumes, however, is the vast underutilization of prone positioning (PP) for patients with severe ARDS. Just over 3 years ago the Proning Severe ARDS Patients (PROSEVA) study investigators showed in their multicenter prospective randomized control trial of 466 (237 prone vs. 229 supine) patients with severe ARDS (defined as a ratio of the partial pressure of arterial oxygen to the fraction of inspired oxygen (Fio_2_) of less than 150 mmHg) that PP definitively reduced mortality by 50% (28-day mortality reduction of 32.8% in supine group to 16.0% in prone group) [3]. An intervention that reduces mortality by 50%, but is only used in 16.3% of appropriate patients, begs the question of *why*. Just imagine the uproar if only 16.4% of patients with CHF with reduced ejection fraction and NYHA III–IV symptoms were prescribed with aldosterone antagonists, a therapy proven to reduce all-cause mortality by 30%, or only 16.4% of patients with moderate or severe COPD were prescribed with long-acting anticholinergics (i.e., tiotroprium), a universally prescribed medication that has only shown statistically significant benefit in reducing exacerbations and not survival; my guess is that these dismal utilization rates would be quickly addressed [4, 5]. Since reviewing the PP literature, I have conversed with colleagues regarding PP’s unpopularity and underutilization, with the majority of their responses focusing on PP’s perceived cumbersomeness, burdensome need for additional human resources, and higher rate of adverse events (i.e., pressure ulcers, accidental extubations, and tracheal tube displacement). Despite this anecdotal majority focus on PP’s onerousness and higher complication rate, scrutiny of the literature suggests otherwise [6–10]. Countless studies, meta-analyses, and reports have continuously shown notions that PP is difficult to initiate, burdensome to maintain, and more apt to cause complications unfounded [11–13]. In fact, Athota et al. reviewed various institutional experiences with PP and highlighted its facility and ease of use, while a recent Cochrane review found no convincing evidence of harm from universal application of PP. Thus, many paramount questions regarding PP’s unfavorable reputation persist with a couple being: (1) why is PP so underutilized in the appropriate setting, and how can we elucidate the causes of this underutilization phenomenon? (2) Prior to making firm conclusions about PP’s efficacy, it would be prudent to conduct multicenter randomized control trials testing PP’s ability to reduce mortality. Why have these studies not been performed? (3) Once the etiologies for PP’s underuse are determined, what interventions can be implemented to improve the widespread adoption and utilization rate of PP, and how can we test the efficacy of these interventions?

## Conclusions

PP has enormous potential to save lives in patients with severe ARDS. Now is the time to focus on ways to address and improve the PP underutilization phenomenon so that more than 16.4% of patients can benefit from this lifesaving intervention.
